# Qualitative smell/taste disorders as sequelae of acute COVID-19

**DOI:** 10.1007/s10072-021-05611-6

**Published:** 2021-09-23

**Authors:** Tommaso Ercoli, Carla Masala, Ilenia Pinna, Gianni Orofino, Paolo Solla, Lorenzo Rocchi, Giovanni Defazio

**Affiliations:** 1 Department of Medical Sciences and Public Health, Institute of Neurology, University of Cagliari, Cagliari, Italy; 2 Department of Biomedical Sciences, University of Cagliari, Monserrato, Italy; 3 Institute of Neurology, Azienda Ospedaliero Universitaria di Cagliari, Cagliari, Italy; 4 Department of Neurology, University of Sassari, Sassari, Italy

**Keywords:** COVID-19, Phantogeusia, Olfactory hallucination, Smell, Taste

## Abstract

**Background:**

Qualitative smell/taste disorders (such as phantosmia, parosmia, phantogeusia, and parageusia) have not yet been fully characterized in patients who had COVID-19, whereas quantitative disturbances (i.e., reduction/loss of smell/taste) have been widely investigated.

**Objective:**

To simultaneously assess the presence of both quantitative and qualitative smell/taste dysfunctions in patients who suffered from COVID-19.

**Methods:**

We enrolled 17 consecutive patients who suffered from COVID-19 over the last 6 months and 21 healthy controls, matched for sex and age. After a negative nasopharyngeal swab, the Sniffin’ Sticks Test and the Taste Strips were used to assess olfactory and taste function, respectively. At the same time, the presence of phantosmia, parosmia, phantogeusia, and parageusia was investigated with a standardized questionnaire.

**Results:**

Qualitative disturbances of smell and/or taste were found in 6/17 (35.3%) patients. Phantosmia was reported in 2/17 (11.8%) patients and parosmia in 4/17 (23.5%). There were no significant differences in smell test scores between patients who reported phantosmia and/or parosmia and patients who did not. Phantogeusia was described in 3/17 (17.6%) patients, and parageusia was identified in 4/17 (23.5%) patients. All tested patients were normogeusic.

**Conclusion:**

Around one-third of patients who recover from COVID-19 may have persistent qualitative dysfunction in smell/taste domains. Detection of phantogeusia in long-term COVID-19 patients represents a further novel finding. Further investigation is needed to better characterize the pathophysiology of phantosmia, parosmia, phantogeusia, and parageusia in patients who had COVID-19.

## Introduction

Olfactory and taste dysfunctions are among the most prevalent symptoms of the acute phase of the coronavirus disease of 2019 (COVID-19) [1, 2]. Their presentation is variable, including total or partial loss of smell (anosmia/hyposmia) and taste (ageusia/hypogeusia), distorted perception of smell/taste (parosmia and parageusia), and perception of an odor or a taste without any concurrent stimulus (phantosmia, also known as olfactory hallucination, and phantogeusia, also known as gustatory hallucination) [3]. Distorted/hallucinatory perceptions of smell/taste are typically highlighted during clinical interview, whereas loss of smell/taste is usually disclosed by objective tests, like the Sniffin’ Sticks Test and the Taste Strips, respectively.

Although reduction/loss of smell/taste during the acute phase of the 2019 novel coronavirus SARS-CoV-2 infection and the following months has been investigated [4, 5], little is known about qualitative smell/taste symptoms [6]. As far as olfactory hallucinations are concerned, only one study evaluated phantosmia long after the acute phase of COVID-19 [7]. On the other hand, parosmia and parageusia have already been described as COVID-19 sequalae, but only a few studies also provided objective measures of olfactory/gustatory function.

Therefore, the pathophysiological mechanisms underlying olfactory/taste dysfunctions after COVID-19 are still debated. To expand current knowledge, in this study, we simultaneously assessed the presence of qualitative smell/taste disturbances and objectively measured smell/taste in patients recovering from COVID-19.

## Methods

Seventeen consecutive patients seen over the last 6 months at the Medical Department of the University Hospital of Cagliari [8, 9] participated into the study. According to the disease severity scoring of World Health Organization [10], COVID-19 manifested in severe form in 1/17 patients (5.8%) that required hospitalization, while 8/17 patients (47.1%) suffered from a moderate form (clinical evidence of pneumonia with SpO2 ≥ 90%), and 8/17 patients (47.1%) reported a mild symptomatology and were treated at home. Patients were examined 63.6 ± 40.8 days (range 8–151 days) after the negative nasopharyngeal swab. Exclusion criteria were history of head or neck trauma [11–13], cognitive impairment and psychiatric conditions interfering with study participation [14, 15], chronic/acute rhinosinusitis, neurological diseases involving smell/taste function [16–18], and systemic diseases related to smell/taste disorders [19].

The Sniffin’ Sticks Test (Burghart Messtechnik, Wedel, Germany) and the Taste Strips (Burghart Messtechnik, Wedel, Germany) were administered to both patients and healthy controls by an examiner who did not know whether the subject complained of qualitative smell/taste disturbances. Seventeen patients were compared with twenty-one healthy controls, matched for sex (10/17 vs. 14/21 females, *p* = 0.74) and age (35.8 ± 17.5 vs. 35.5 ± 11.1, *p* = 0.73). Healthy controls were from a pool of healthy subjects that had been tested by the Sniffin’ Sticks Test and the Taste Strips before the pandemic (in the first semester of 2019). Full description of the methodology of smell/taste tests is provided elsewhere [20–22]. In brief, the Sniffin’ Sticks Test takes into account three different parameters: odor threshold (OT), odor discrimination (OD), and odor identification (OI). A total score (TDI, resulting from the sum of the OT, OD, and OI scores) > 30.5, ≤ 30.5, and ≤ 16.5 indicates normosmia, hyposmia, and anosmia, respectively. Taste Strips assess four taste modalities (sweet, bitter, sour, and salty) using four concentrations for each modality. Taste Strips total score ranges from 0 to 16, with a score < 9 indicating hypogeusia.

After performing objective smell/taste tests, patients and controls were also asked about smoking history, as well as by ongoing and prior occurrence of phantosmia, parosmia, phantogeusia, and parageusia by a standardized interview characterized by a brief description/explanation of qualitative alterations of smell/taste [22]. No healthy control reported ongoing or prior occurrence of distorted/hallucinatory perception of smell/taste.

Statistical analysis was performed with the Stata 11.0 package (StataCorp LP, College Station, TX). Data were expressed as mean ± standard deviation unless otherwise indicated. Differences between groups were examined by chi-square test, Fisher’s exact test, or the Mann-Whitney U test as appropriate [23–25]. For all analyses, significance was set at 0.05.

## Results

Patients were examined 63.6 ± 40.8 days (range 8–151) after the negative nasopharyngeal swab. Qualitative disturbances of smell and/or taste starting during the acute phase of the 2019 novel coronavirus SARS-CoV-2 were found in 6/17 patients (35.3%) 99 ± 30.1 days after the negative swab. The remaining 11/17 patients did not experience any qualitative symptoms of smell/taste either during the acute phase of COVID-19 or on examination performed 44.3 ± 32.3 days after the negative swab. The time elapsing between the negative swab and examination was significantly shorter in the 11 patients without qualitative smell/taste disturbances after COVID-19 (*p* = 0.003). No patients reported olfactory or gustatory disturbances before COVID-19.

Among the six patients who reported qualitative smell and/or taste disturbances, both domains were affected in three patients, the smell domain alone in two patients, and the taste domain alone in one patient (Table 1). Smell/taste disturbances were found in 1/1 patient who had the severe form of COVID-19, in 4/8 patients who suffered from the moderate form, and in 4/8 patients who had the mild form (*p* = 0.94), likewise there was no difference among smokers and ever smokers in the COVID-19 group (*p* = 0.62).

**Table 1:** Qualitative disturbances of smell and/or taste in six patients who had COVID-19. All patients were normogeusic according to the Taste Strip test total score

Patients	Phantosmia	Parosmia	Anosmia	Hyposmia	Phantogeusia	Parageusia
n. 1	N	Y	-	Y	Y	Y
n. 2	Y	Y	-	Y	N	Y
n. 3	N	Y	-	-	N	N
n. 4	N	Y	-	-	N	N
n. 5	N	N	-	-	Y	Y
n. 6	Y	N	Y	-	Y	Y

Qualitative smell disturbances were reported by five patients. Phantosmia was reported by one patient (5.8%) who was anosmic and described olfactory hallucination as a strong and disgusting odor (e.g., burning smell); parosmia was reported by 3/17 patients (17.6%), of whom one was hyposmic and two were normosmic on the Sniffin’ Sticks Test; a further hyposmic patient reported both phantosmia (defined as a slight and pleasant smell) and parosmia. All patients with parosmia described an unpleasant distorted perception of smell (e.g., smell of garbage/rotten food while eating).

Qualitative taste abnormalities were reported by four patients. Both phantogeusia and parageusia were found in 3/17 patients (17.6%): one patient described the gustatory hallucination as a strong and disgusting taste, while the other two patients experienced a slight and pleasant flavor (e.g., sweet taste without a meal). Parageusia alone was identified in one patient (5.8%). Three patients described parageusia as an unpleasant distorted perception of gustatory sense (e.g., fuel/iron and plastic taste while eating), while one patient described the misperception as slight and pleasant.

**Fig 1.**
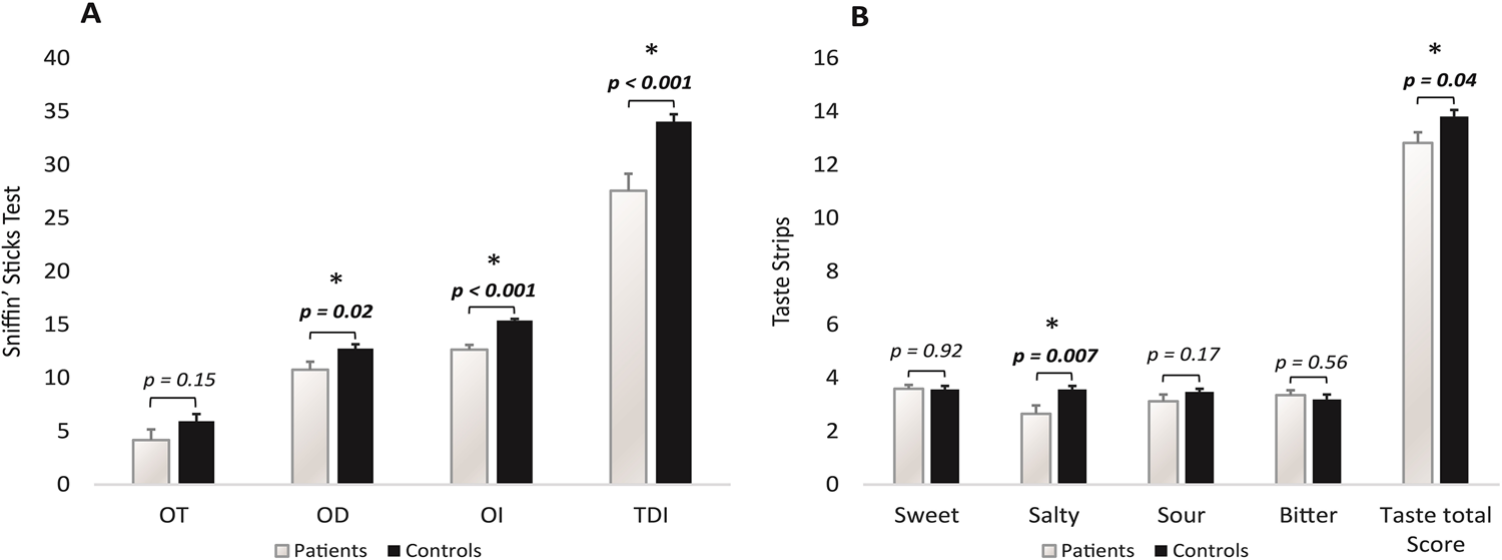
Sniffin’ Sticks Test scores (**A**) and Taste Strips scores (**B**) of the 17 patients who had COVID-19 and the 21 healthy controls.

In comparison to controls, patients showed a significant decrease in olfactory function as assessed by the Sniffin’ Sticks Test (Fig. 1) (TDI score: 34.1 ± 3.2 vs. 27.6 ± 6.7, *p* < 0.001). The two groups were similar in terms of sex (10 women and 7 men vs. 14 women and 7 men, *p* = 0.51), age (35.8 ± 17.5 vs. 35.5 ± 11.1, *p* = 0.95), and smoking history (11 non-smokers and 6 ever smokers vs. 15 non-smokers and 7 ever smokers, *p* = 0.46). There was no significant difference in the Sniffin’ Sticks Test between the five patients who reported phantosmia and/or parosmia and the twelve patients who did not (TDI score: 25.5 ± 7.1 vs. 28.4 ± 6.5, *p* = 0.43). According to the cut-off from the Sniffin’ Sticks Test, 1/17 (5.8%) patient was anosmic and 8/17 (47.1%) patients were hyposmic and 8/17 (47.1%) patients were normosmic. Hyposmia/anosmia was found in 3/5 patients who reported phantosmia/parosmia and in 6/12 patients who did not (Fisher’s test, *p* = 1). Stratifying the results of the Sniffin’ Sticks Test by OT, OD, and OI in the five patients who reported phantosmia/parosmia and the twelve patients who did not, no significant differences were detected between the two groups (OT score: 2.6 ± 2.1 vs. 4.8 ± 4.7, *p* = 0.32; OD score: 11.2 ± 3.9 vs. 10.6 ± 2.8, *p* = 0.71; OI score: 11.8 ± 2.3 vs. 13 ± 1.5, *p* = 0.22). Comparing the three patients who had parageusia/phantogesia associated to hyposmia/anosmia to the three patients who were normosmic yielded non-significant differences in OD (9 ± 3.5 vs. 13.7 ± 1.5, *p* = 0.1) and OI (10.7 ± 2.3 vs. 13.7 ± 0.6, *p* = 0.09).

Although the total score from Taste Strip test was slightly but significantly lower in COVID-19 patients than in controls (Fig. 1), Taste Strip values from COVID-19 patients were all in the range of normal taste.

## Discussion

In this study, we documented the persistence of qualitative disturbances of smell and/or taste in 35% of patients who had COVID-19 acute infection. Compared to age- and sex-matched healthy controls, our patients also showed a significant persistent decrease in olfactory but not taste function. However, patients who reported phantosmia/parosmia and those who did not showed similar reduction in the olfactory function.

Persistent phantosmia has recently been described as a long-term symptom in a number of patients, while data on persistent parosmia are sparse [7]. Persistent parageusia has been reported in about 10% of patients 60.3 ± 13.6 days after COVID-19 acute infection while gustatory hallucination has never been described as sequalae of COVID-19 [4]. Therefore, this is the first report of phantogeusia as a persistent symptom of the 2019 novel coronavirus SARS-CoV-2 infection. By contrast, loss/reduction of smell/taste has been already investigated in patients who suffered from COVID-19, with results indicating only a partial improvement of smell/taste disorders 40 days after diagnosis in about one-third of patients [26, 27].

Since phantosmia/parosmia may be associated with olfactory loss [28], we assessed olfactory detection ability with the Sniffin’ Sticks Test that evaluates three parameters (i.e., OT, OD, and OI) probably reflect different pathways of activation in the olfactory system. OT is considered to be related to nasal epithelium and individual differences in the nasal cavity, while OD and OI are usually associated to central pathways connecting the orbitofrontal cortex, the piriform cortex, and the amygdala [29]. We found a significant decrease of quantitative smell function in the group of patients who had COVID-19 even though no significant differences in OT, OD, and OI were observed between patients who reported qualitative smell disturbances and those who did not. This does not support quantitative smell impairment as a significant factor contributing to phantosmia/parosmia in our patients. Even though olfactory loss and phantosmia/parosmia may coexist [28], our finding is nevertheless in line with the observation that qualitative smell disorders may also occur among subjects with normal olfactory function [30–32]. The lack of taste loss (as assessed by Taste Strips test) in our patients with qualitative gustatory disturbances suggests that also parageusia/phantogeusia may be associated with a normal taste system. Since parageusia/phantosmia seems to be a central phenomenon, we checked whether OD and OI scores (the ones associated to central pathways) were more reduced in the patients who had parageusia/phantosmia associated with quantitative smell disturbances. Probably due to the very low number of study participants, we were not able to find any significant statistical power toward such finding.

The pathophysiology of smell and taste impairment secondary to the 2019 novel coronavirus SARS-CoV-2 infection remains to be elucidated [33]. However, several previous studies proposed pathophysiological hypotheses of olfactory/smell dysfunctions after COVID-19. Anosmia was related to olfactory bulb atrophy on MRI in a patient with COVID-19 [34], and similar findings have been confirmed in other studies [35, 36]. Interestingly, elevated levels of cytokine were found in the olfactory epithelium of COVID-19 patients suggesting that the direct inflammation of the olfactory tract could play a crucial role in the development of sensory loss [37].

Hyposmia may be an early known pre-motor symptom in Parkinson’s disease. This raises the possibility of a contribution of COVID-19 in the development of this multifactorial condition [38]. Although few cases of parkinsonism have been described after 2019 novel coronavirus SARS-CoV-2 infection, the causal relationship between COVID-19 and the development of Parkinson’s disease is not yet supported by robust clinical/etiopathogenic evidence [39, 40].

This study has some limitations. We did not objectively evaluate taste/smell function during the acute phase of the 2019 novel coronavirus SARS-CoV-2. We also did not evaluate other clinical/behavioral long COVID symptoms like fatigue, breathlessness, and muscle and body aches. Therefore, we did not know whether our patients with persistent qualitative smell/taste disturbances could be considered as “Long COVID” patients, according to the definition of the National Institute for Health and Care Excellence [6]. The small sample size and the low associated study power reduced the possibility to assess the prevalence of qualitative smell and taste disorders as well as their association with the olfactory and taste objective impairment. We also could not provide any magnetic resonance imaging data about olfactory-related areas of patients who had anosmia/hyposmia during the acute COVID-19 phase. Nevertheless, our findings shed more light on the possible long-term neurological sequalae of COVID-19. Further research is needed in order to better evaluate the frequency of phantosmia, parosmia, phantogeusia, and parageusia in patients who had COVID-19, and to better characterize their pathophysiology.
